# Kawasaki disease with shock as the primary manifestation: How to distinguish from toxic shock syndrome?: A case report and literature review

**DOI:** 10.1097/MD.0000000000039199

**Published:** 2024-08-02

**Authors:** Weijuan Wang, Huixia Wang, Huijiao Wang, Jun Cheng

**Affiliations:** a Department of Pediatric Intensive Care Unit, Northwest Women’s and Children’s Hospital, Xi ‘an, Shaanxi Province, China.

**Keywords:** Kawasaki disease, Kawasaki disease shock syndrome, polyserous effusion, pulmonary consolidation, toxic shock syndrome

## Abstract

**Rationale:**

Kawasaki disease (KD) is a vasculitis syndrome of small to medium-sized arteries that has typical clinical characteristics such as fever, rash, cervical lymphadenopathy, conjunctivitis, and mucosal changes. Cardiac manifestations, including coronary artery aneurysms, myocarditis, myocardial infarction, and sudden cardiac death, are the most serious complications observed in KD. On rare occasions, it may accompanied with reduced organ perfusion due to systolic hypotension, a condition known as Kawasaki disease shock syndrome (KDSS). KDSS is a serious complication that can be presented to the emergency department as an initial feature when typical clinical symptoms of KD have not be detected.

**Patient concerns:**

We report the case of a 12-year-old boy admitted with prolonged fever, bilateral non-purulent conjunctivitis, and signs of shock such as hypotension and tachycardia. Laboratory findings showed elevated inflammatory markers, hypoalbuminemia, and sterile pyuria. He was initially treated with intravenous cefotaxime and vancomycin considering the possible diagnosis of toxic shock syndrome, while the treatment was not effective. Subsequent chest computerized tomography and ultrasound identified pulmonary consolidation and polyserous effusion. Echocardiography revealed mild biatrial dilatation and mild valvular regurgitation with preserved left ventricular function.

**Diagnosis:**

After a multidisciplinary consultation, a diagnosis of KDSS was made.

**Interventions:**

To prevent coronary artery lesions and other severe complications, the patient immediately received immunoglobulin, corticoid, and acetylsalicylic acid.

**Outcomes:**

Soon afterwards, he showed significant improvement, with the temperature dropped to normal and hypotension corrected about 24 hours post-intravenous immunoglobulin therapy. Polyserous effusions also disappeared before discharge. Follow-up echocardiography revealed normal results.

**Lessons:**

Clinicians should maintain a high index of suspicion for KD and consider pulmonary involvement and polyserous effusions as potential complications. For children with KD, any symptoms pointing to infection should be carefully considered. When there is no etiologic evidence, antibiotics should be used with caution. Our case also highlights the importance of considering KDSS as a differential diagnosis in children presenting with prolonged fever and shock. Early recognition, timely treatment, and close monitoring are key to preventing severe complications and ensuring favorable outcomes in patients with KDSS.

## 1. Introduction

Kawasaki disease (KD), also known as mucocutaneous lymph node syndrome, is an acute rheumatological illness mostly affecting children between 6 months and 5 years of age.^[1]^ But less commonly, it can affect children of ages beyond this range.^[2]^ The diagnosis of KD involves identifying the presence of a persistent febrile state persisting for a minimum duration of 5 days, concomitant with the presence of no less than 4 characteristic manifestations from the following diagnostic criteria^[3]^: non-purulent conjunctivitis bilaterally exhibiting sparing of the limbal region; oropharyngeal mucosal changes encompassing erythematous fissured lips, a tongue displaying a strawberry-like appearance, or diffuse erythema within the oropharynx; a generalized rash of indeterminate nature; edema and erythema affecting the extremities; and unilateral cervical lymphadenopathy.^[3–5]^ However, a small subset of children with KD may exhibit only 2 or 3 of these typical KD symptoms, making early detection of KD difficult. In this case, we can only look for clues of “incomplete Kawasaki disease” from follow-up laboratory tests, such as increased C-reactive protein (CRP), increased erythrocyte sedimentation rate, anemia, increased platelet, hypoalbuminemia, aseptic pyuria, increased alanine aminotransferase, and so on. Kawasaki disease shock syndrome (KDSS) was first described by Kanegaye et al^[4]^ in 2009, which is characterized by the simultaneous presence of KD symptoms and features of shock. The diagnosis of KDSS can be made if the sustained presence of any of the following conditions caused the treating clinicians to initiate volume expansion, infusion of vasoactive agents, or transfer to an intensive care setting: systolic hypotension for age (infants 0–28 days of age, <60 mm Hg; infants 1–12 months of age, <70 mm Hg; children 1–10 years of age, <70 + [2 × age] mm Hg; youths > 10 years of age, ≤90 mm Hg3,4), a decrease in systolic blood pressure from baseline of ≥20%, or clinical signs of poor perfusion (tachycardia, prolonged capillary filling time, cool extremities, diminished pulses, oliguria, or mental status changes not accounted for by other conditions such as fever or ambient temperature) regardless of measured blood pressure.^[4]^ The specific etiology of profound hypotension in KDSS is still not fully understood, but it is hypothesized to be related to ongoing vasculitis, leakage of capillaries, impaired myocardial function, and dysregulation of cytokines at a systemic level. For children exhibiting signs of shock at an early stage of KD before enough typical clinical symptoms presented, the diagnosis and treatment will be challenging. While KDSS can affect multiple organs, polyserous effusions are not common. There are only reports describing KDSS complicated by the presence of bilateral pleural effusion.^[6,7]^

## 2. Case presentation

### 2.1. Patient information

A 12-year-old boy was brought to our hospital in November 2023, with complaint of fever accompanied by headaches that had persistent for 4 days. The boy had been in good health until 4 days ago when he developed a fever which was, according to his parents, high-grade, with the highest temperature 39.3°C, and almost continuous with only mildly relieved by paracetamol. On second day of illness, he developed generalized maculopapular rashes on the body. He was taken to a local hospital for treatment with these complaints. Laboratory testing indicated leukocytosis with increased neutrophils. Thus he was treated with antibiotics (erythromycin for 1 day and azithromycin for 2 days). The antibiotics and antipyretics given in the local hospital could not adequately control his fever, and his condition progressively deteriorated. Therefore, he was referred to our center on day 4 of the course. Apart from a history of several respiratory infections, he was in good health and had no history of special medication. No special family history was found too. The family raised no pets. Vaccinations, including the COVID-19 vaccine, were carried out according to the national plan. He lived in a good environment, and the water quality met the national standard.

### 2.2. Clinical findings

The child was presented to our hospital in the emergency department. He was 1.53 m tall and weighed 40 kg. He was weak and unconscious at a glance. Follow-up examination revealed that the child exhibited signs of shock: a prolonged capillary refill time of more than 4 seconds, weak peripheral pulses, hypotension, and tachycardia. The blood pressure, temperature, oxygen saturation, and pulse rate during the initial assessment of the patient at the emergency department were 87/56 mm Hg, 38.5°C, 97%, and 128 beats per minute, respectively. Abdomen examination revealed slight tenderness in the periumbilical area with no hepatosplenomegaly. To address the condition, a single bolus of fluid (20 mL/kg) were administered rapidly, then blood investigations including blood cultures were conducted. Subsequently, the child was transferred to the pediatric intensive care unit for meticulous surveillance and monitoring. Table 1 presents the noteworthy results of the comprehensive blood tests conducted, providing valuable insights into the patient’s condition.

**Table 1:** Some laboratory parameters during hospitalization.

Blood investigation [reference range]	Day 1	Day 2	Day 3	Day 4	Day 5	Day 7	Day 10
CRP (mg/dL) [0–10]	83.80	87.89	81.17	34.34	18.00	8.55	<6
hsTnT (ng/mL) [0–2.41]	-	4.4	-	-	-	-	-
BNP (pg/mL) [0–135]	288.40	927.50	879.00	784.50	160.50	-	19.40
Hemoglobin (g/dL) [118–156]	10.7	10.5	10.6	10.9	11.4	12.6	13.0
White blood cells (×10^9^) [4.3–11.3]	15.33	15.62	10.41	8.84	9.28	12.16	5.82
Neutrophil (×10^9^) [1.6–7.8]	12.54	11.8	7.75	5.10	3.71	4.88	3.31
Lymphocyte (×10^9^) [1.5–4.6]	1.54	1.62	1.95	2.74	4.08	5.87	1.77
Platelet (×10^9^) [167–453]	218	190	242	301	316	361	326
Procalcitonin (ng/mL) [<0.05 ng/mL]	0.49	0.43	0.22	0.10	0.07	-	-
Albumin (g/L) [39–54]	27.28	27.56	30.61	31.63	34.19	35.76	39.87
ESR (mm/1h) [0–15]	-	13	-	-	15	-	10
IL-6 (pg/mL) [<9.1]	257.05	45.58	1.50	-	-	-	-
IL-10 (pg/mL) [<8.2]	62.9	-	-	-	-	-	-
TNF-α (pg/mL) [<15.3]	5.74	-	-	-	-	-	-
D-dimer (µg/mL) [0–2.0]	0.77	-	1.23	1.45	1.29	-	-
Ferritin (ng/mL) [21.81–274.66]	118.91	105.98	113.39	129.15	-	-	-

BNP = brain natriuretic peptide, CRP = C-reactive peptide, ESR = erythrocyte sedimentation rate, hsTnT = hypersensitive troponin T, IL-10 = interleukin 10, IL-6 = interleukin 6, TNF α = tumor necrosis factor α.

### 2.3. Diagnostic assessment and therapeutic intervention

**Figure 1.**
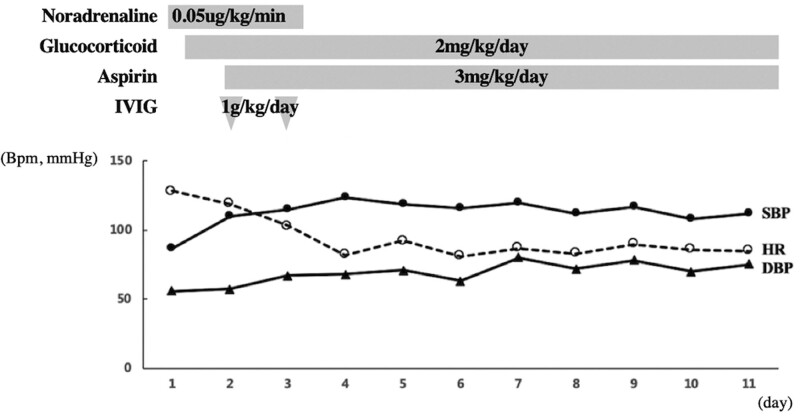
Clinical course in the acute phase of KDSS. DBP = diastolic blood pressure, HR = heart rate, IVIG = intravenous immunoglobulin, KDSS = Kawasaki disease shock syndrome, SBP = systolic blood pressure.

On further examination, he was found to have bilateral non-purulent conjunctivitis, along with edematous limbs. The initial echocardiography did not show any diastolic dysfunction, and the systolic function was normal too, with an ejection fraction of 60%. The laboratory results indicated leukocytosis with increased neutrophils and positive CRP, while erythrocyte sedimentation rate was normal. The COVID-19 acid test was negative at the time of hospitalization. Since the inflammatory markers were very high, meanwhile, the child presented with shock and maculopapular rashes, the preliminary diagnosis of toxic shock syndrome (TSS) was considered. Thus, we initiated anti-infection treatment with intravenous cefotaxime and vancomycin. Continuous infusion of norepinephrine was also adopted because of persistent hemodynamic instability and hypotension. Corticoid therapy was initiated because of capillary leak and shock. Figure 1 shows clinical course in the acute phase of KDSS. Initial work-up showed hypoalbuminemia with a decreased albumin level of 27.28 g/L. To improve this, a 20% albumin transfusion was administered at a dosage of 1 g/kg of the child’s weight in 2 separate days. Additionally, the initial chest X-ray revealed bronchopneumonia. Increased leukocytes (36.46/HFP) in urine sediment was also found. There was a significant elevation of brain natriuretic peptide. No sign of other organ dysfunction was found.

**Figure 2.**
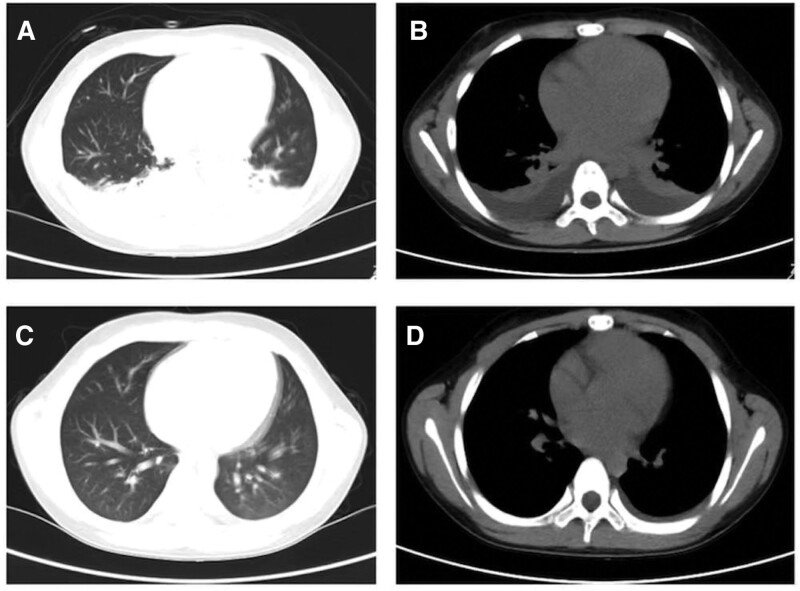
Lung consolidation and pleural effusion on CT. (A, B) Chest CT during the acute phase of disease revealed lung consolidation and pleural effusion. (C, D) Lung consolidation and pleural effusion gradually absorbed after treatment. CT = computerized tomography.

**Figure 3.**
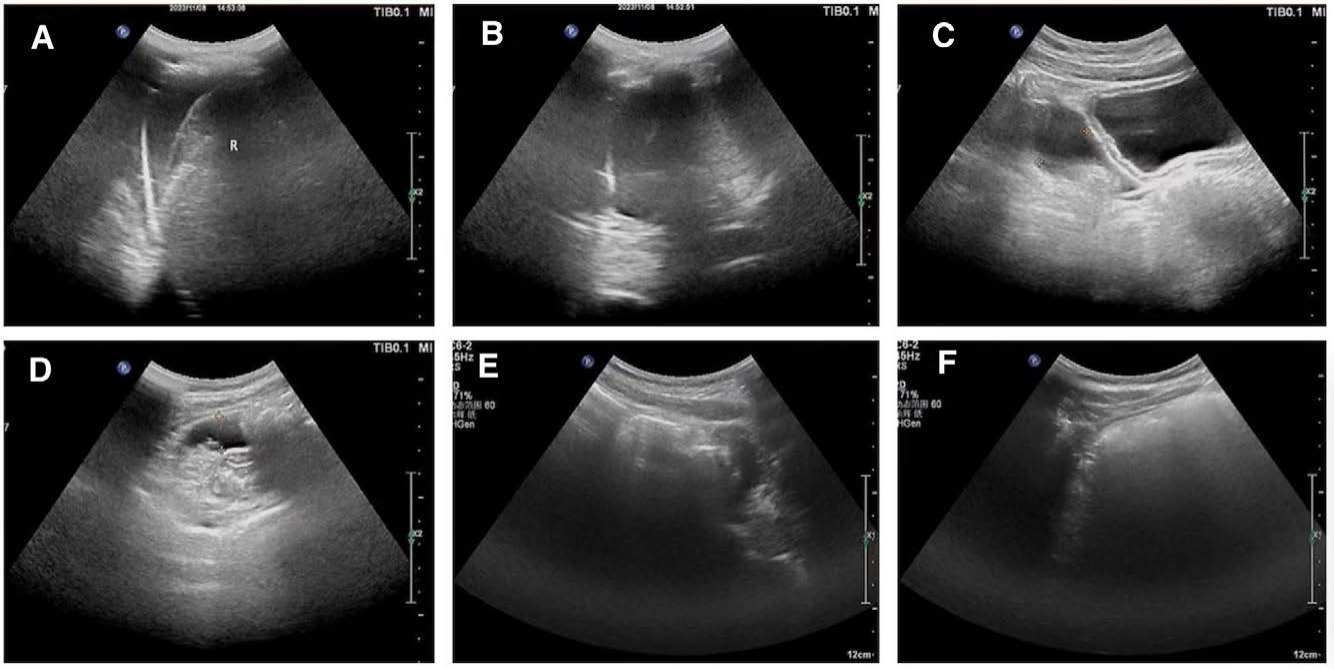
(A–D) Ultrasound during the acute phase of disease revealed bilateral pleural effusion, ascites, and pelvic effusion. (E, F) Ultrasound showed a gradual resolution of the peritoneal effusion in response to therapy.

On the second day of admission, the child continued to experience a persistent high-spiking fever, with the highest temperature 39.2°C. The CRP level elevated even higher in spite of receiving powerful intravenous antibiotic treatment. Chest computerized tomography showed lung exudation and consolidation (Fig. 2A, B). An echocardiography was repeated what revealed mild biatrial dilatation (left atrial systolic diameter 40 mm, right atrial systolic diameter 39 mm) and mild valvular regurgitation with preserved left ventricular function (ejection fraction 59%). The left and right ventricles and other structures of the heart were not abnormal. No pericardial effusion was found. Coronary artery widening was not observed. The internal diameter of the left main coronary artery was 2.5 mm with a *Z* value of −1.25, while the left anterior descending coronary artery diameter 1.9 mm (*Z* = −1.58), the right main coronary artery diameter 2.3 mm (*Z* = −0.94). Furthermore, an ultrasound examination of the abdomen and chest was conducted, which identified moderate amount of bilateral pleural effusion and mild ascites, as well as mild free fluid in the pelvic region (Fig. 3A–D). About 10 mL of hydrothorax was collected and analyzed, indicating transudate. The results of EB virus nucleic acid test, COVID-19 acid test, sputum culture, hydrothorax culture, and blood culture were all negative. The mycoplasma antibody test, the cytomegalovirus antibody test, and the tuberculin test were all negative. A multidisciplinary consultation was held. According to the clinical manifestation, features, and laboratory examination results (fever lasting for 5 days, bilateral non-purulent conjunctivitis, edematous limbs, elevated blood leukocyte count and CRP, sterile pyuria, hypotension, and tachycardia), the patient was diagnosed with KDSS on the basis of incomplete KD. The lung consolidation was determined to be a manifestation of KDSS in the lung, rather than TSS complicated by a pulmonary bacterial infection. To address this condition, high-dose intravenous immunoglobulin (IVIG) was administered. Considering the large weight (≧20 kg) of the child, we used a regimen of 1 g of immunoglobulin per kilogram of body weight per day for 2 consecutive days to reduce the occurrence of adverse reactions such as hemolysis. This medication regimen was also based on our country’s expert consensus on the diagnosis and treatment of KD. Additionally, acetylsalicylic acid (3 mg/kg/d) was started to prevent thrombosis and for anti-inflammation. The reason we used low-dose aspirin was that the child’s symptoms of KD were atypical and our initial diagnosis was not so definitive. In addition, large doses of aspirin may cause adverse drug reactions. Careful monitoring of the fever was implemented as part of the management plan. The patient experienced a significant improvement in the condition, with the temperature dropped to normal and hypotension corrected about 24 hours post-IVIG therapy. Norepinephrine was discontinued after a 2-days usage, which indicated that the hemodynamics was stabilized and the shock was reversed. On the fifth day of admission, the diagnosis was supported by the observation of desquamation (skin peeling) on the hands and feet. Eight days after admission, ultrasound examination of the abdomen showed a gradual resolution of the peritoneal effusion (Fig. 3E, F). Soon afterwards, chest computerized tomography also showed a significant absorption of inflammation in the lung (Fig. 2C, D), indicating a positive response to treatment. Antibiotic therapy was stopped after the blood culture result returned negative. Eleven days after admission, he was discharged from the hospital in good condition. He continued to take aspirin at a dosage of 3 mg/kg/d as instructed by doctor. Follow-up visits were scheduled at 2 and 6 weeks after discharge. In order to avoid the occurrence of Reye’s syndrome, the child was instructed to receive influenza vaccine during the epidemic season and take good protection to avoid respiratory infection.

### 2.4. Follow-up and outcomes

To monitor the possibility of cardiac complications associated with KD, the patient was scheduled for follow-up visits at 2 and 6 weeks. Fortunately, echocardiography performed during these visits revealed normal results. As a result, the use of acetylsalicylic acid was discontinued after 6 weeks since the echocardiography findings remained normal.

In summary, the patient’s condition improved dramatically, with fever subsided, hemodynamics stabilized, shock reversed. Follow-up echocardiography revealed all normal results, leading to the cessation of acetylsalicylic acid medication after 6 weeks.

## 3. Discussion

KD, which was first described in 1967 by Dr Tomisaku Kawasaki, is diagnosed frequently in the Asian pediatric populations. Cardiac manifestations, including coronary artery aneurysms, myocarditis, myocardial infarction, and sudden cardiac death, are the most serious complications observed in KD. However, KD can also present in the form of KDSS. Since KD is rarely seen in anyone over 9 years of age,^[8]^ after the peak age of onset, it is not often considered and is probably underdiagnosed to some degree. Interestingly, age >10 years has been reported as one of the risk factors for progression to KDSS.^[9]^ It is crucial to be cognizant of the potential occurrence of KDSS, since prompt recognition and management are necessary to ensure optimal patient outcomes.^[3]^

Although the specific etiology of profound hypotension in KDSS is still not fully understood, there are hypotheses supporting vasculitis with capillary leakage, myocardial function defect, and dysregulation of cytokines at a systemic level, resulting in cardiogenic and/or distributive shock.^[4,10]^ In our case, the boy had severe vascular leakage and hypoalbuminemia, with pleural effusions and ascites. In our opinion, the shock was probably due to a significant inflammatory state and vascular leakage, as no sign of remarkable cardiac dysfunction on echocardiography showed. Natterer et al^[6]^ also described 3 cases of KDSS present with normal myocardial function. However, the significant elevation of brain natriuretic peptide still suggested some myocardial inflammation. Undeniably, myocardial dysfunction in our case might have been underestimated own to timely application of vasoactive agonists, as well as subsequent appropriate management of KDSS.

In our case study, a 12-year-old boy exhibited persistent fever, bilateral non-purulent conjunctivitis, edematous limbs, unresponsive shock, low levels of albumin, significantly elevated levels of CRP. Despite the presence of these clinical indicators, the diagnosis of KDSS was not definitively confirmed until a subsequent phase of the illness. The delayed diagnosis is due to the considerable overlap in clinical features between TSS and KDSS.

For children with KD, multiple systems including the respiratory system can be affected.^[11]^ Common pulmonary manifestations mainly include bronchopneumonia, hydropneumothorax, and pleural effusion. A few studies have reported that KD may appear secondary to lung consolidation, which often occurs due to Streptococcus, Staphylococcus, Mycoplasma, EB virus, coronavirus, or parvovirus infection.^[12]^ Thus, in the current case, it was essential to identify whether lung consolidation appeared secondary to KDSS or TSS.

Considering that, the blood cultures were consistently negative, the transudate did not contain bacterial infection, moreover, lung consolidation appeared during KDSS progression. Thus, pathological changes observed in the lung were considered to be related to KD. In addition, cardiac valve regurgitation, which was seen in our case might be another discriminating factor between KDSS and TSS, since previous studies have found that echocardiographic abnormalities including cardiac valve regurgitation were considerably more common among patients with KDSS.^[13,14]^

In terms of treatment, despite fluid resuscitation and maintenance of hemodynamic stability are similar, the course of disease evolution between KDSS or TSS are different. Generally speaking, multiple organ dysfunction in KDSS was less severe and mostly transient. Meanwhile, shock associated with KD can be controlled relatively easily by vasoactive agent. On the contrary, once the blood pressure drops in septic shock or TSS, the condition is often very critical, the incidence of multiple organ failure is high, and the mortality is significantly increased. Specific antibiotics and glucocorticoid are used to treat TSS, which still leads to high mortality rates (up to 44%) after treatment.^[15]^ For KDSS, IVIG should be administered as soon as possible to achieve a good prognosis, while the use of antibiotics is not required. When unusual pulmonary changes similar to bacterial infection appear, KDSS and TSS should be identified based on monism. For our case, according to the principles of KD therapy, consolidation was absorbed rapidly, and the course of treatment was much shorter than the cure course of consolidation in the bilateral lungs caused by a bacterial infection, which further confirmed the diagnosis of KD-related pulmonary changes.

Our case highlights that children with KD may present with a toxic shock-like illness. It is essential to promptly recognize and remain vigilant for the manifestation of KDSS. Early detection enables timely initiation of treatment, which can have a profound impact on patient outcomes.^[10,16]^

According to previous literature reports, pleural effusion and pericardial effusion frequently present in KDSS. Gamez-Gonzalez et al^[17]^ collected and analyzed data from 103 patients with KDSS. According their data pericarditis and pleural effusion observed in 20% and 50% of the patients, respectively.^[17]^ A report by Kim et al^[18]^ also described a child with incomplete KD, for whom cardiac tamponade and hemorrhagic pleural effusion were the initial clinical manifestations. However, polyserous effusion with ascites is not that common in KDSS. The mechanism of this difference cannot be explained, and further research is needed.

## 4. Conclusions

Clinicians should maintain a high index of suspicion for KD and consider pulmonary involvement and polyserous effusions as potential complications. This case also highlights the importance of considering KDSS as a differential diagnosis in children presenting with prolonged fever and shock. KDSS can present with normal myocardial function which suggest that the shock was probably due to a significant inflammatory state and vascular leakage. Early recognition and timely treatment are key to preventing severe complications and ensuring favorable outcomes in patients with KDSS. Despite the considerable overlap in clinical features between TSS and KDSS, etiological test results, echocardiography, and response to fluid resuscitation or vasoactive drugs can be used to distinguish the 2 diseases. Further research and awareness are needed to better understand the underlying mechanisms of KD, its varied presentations, and the optimal management strategies. By expanding our knowledge and improving diagnostic capabilities, we can enhance the care provided to children affected by this challenging disease.

## Author contributions

**Data curation:** Weijuan Wang, Huixia Wang.

**Writing – original draft:** Weijuan Wang.

**Supervision:** Huijiao Wang.

**Conceptualization:** Jun Cheng.
